# Circulating Autoantibody Profiling Identifies LIMS1 as a Potential Target for Pathogenic Autoimmunity in pathologic Myopia

**DOI:** 10.1016/j.mcpro.2024.100783

**Published:** 2024-05-09

**Authors:** Jiao Qi, Hao Li, Yu Du, Yun Liu, Wenwen He, Jiaqi Meng, Ling Wei, Keke Zhang, Yi Lu, Xiangjia Zhu

**Affiliations:** 1 Eye Institute and Department of Ophthalmology, Eye & ENT Hospital, Fudan University, Shanghai, People’s Republic of China; 2 NHC Key Laboratory of Myopia and Related Eye Diseases, Key Laboratory of Myopia and Related Eye Diseases, Chinese Academy of Medical Sciences, Shanghai, People’s Republic of China; 3 Shanghai Key Laboratory of Visual Impairment and Restoration, Shanghai, People’s Republic of China; 4 State Key Laboratory of Medical Neurobiology, Fudan University, Shanghai, People’s Republic of China; 5 Department of Ophthalmology, Zhongshan Hospital, Fudan University, Shanghai, People’s Republic of China; 6 MOE Key Laboratory of Metabolism and Molecular Medicine, Department of Biochemistry and Molecular Biology, School of Basic Medical Sciences, Fudan University, Shanghai, People’s Republic of China

**Keywords:** high myopia, pathologic myopia, myopic macular degeneration, HuProt array, anti-LIMS1 autoantibody, autoimmunity

## Abstract

High myopia is a leading cause of blindness worldwide, among which pathologic myopia, characterized by typical myopic macular degeneration, is the most detrimental. However, its pathogenesis remains largely unknown. Here, using a HuProt array, we first initiated a serological autoantibody profiling of high myopia and identified 18 potential autoantibodies, of which anti-LIMS1 autoantibody was validated by a customized focused microarray. Further subgroup analysis revealed its actual relevance to pathologic myopia, rather than simple high myopia without myopic macular degeneration. Mechanistically, anti-LIMS1 autoantibody predominantly belonged to IgG1/IgG2/IgG3 subclasses. Serum IgG obtained from patients with pathologic myopia could disrupt the barrier function of retinal pigment epithelial cells *via* cytoskeleton disorganization and tight junction component reduction, and also trigger a pro-inflammatory mediator cascade in retinal pigment epithelial cells, which were all attenuated by depletion of anti-LIMS1 autoantibody. Together, these data uncover a previously unrecognized autoimmune etiology of myopic macular degeneration in pathologic myopia.

## Highlights

• Serological autoantibody profiling of high myopia detected by HuProt array.
• Serum anti-LIMS1 autoantibody as a potential biomarker for pathologic myopia.
• Anti-LIMS1 disrupts the barrier function of retinal pigment epithelial cells.
• Anti-LIMS1 triggers a pro-inflammatory cascade in retinal pigment epithelial cells.
• Autoimmune etiology of myopic macular degeneration in pathologic myopia.

## Graphical Abstract

**Figure:**
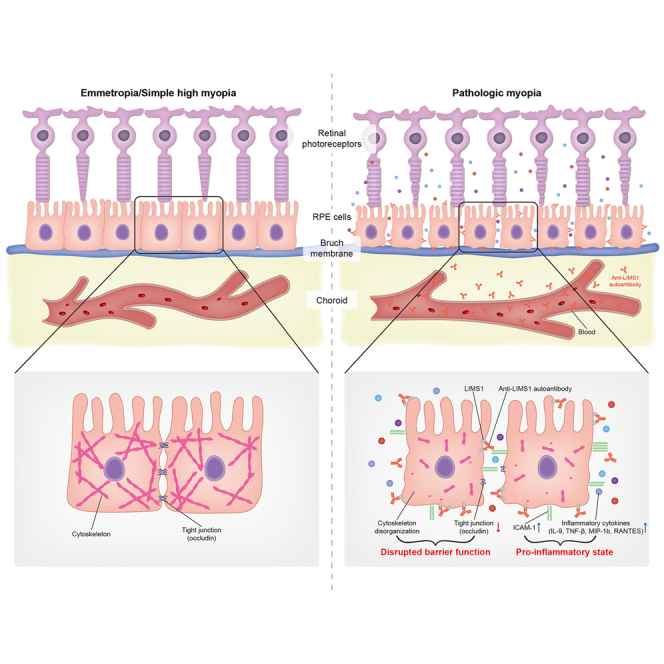

## In Brief

An unbiased human proteome array followed by a customized focused microarray identified serum anti-LIMS1 autoantibody as the high myopia-related autoantibody. Further subgroup analysis revealed its actual relevance with pathologic myopia, rather than simple high myopia without typical myopic macular degeneration. Mechanistic exploration unveiled a detrimental effect of anti-LIMS1 autoantibody on retinal pigment epithelial cells. These observations together support that serum anti-LIMS1 autoantibody could serve as a potential biomarker for pathologic myopia, and uncover a previously unrecognized autoimmune etiology.

High myopia, with surging prevalence in recent decades, has become a major public health concern that affects 400 million people worldwide (1). Distinctly different from low and moderate myopia, high myopia is sight-threatening, with a higher risk of complications including cataracts, neuropathy, maculopathy, retinal detachment, and so on. (2, 3, 4). Thereinto, pathologic myopia, characterized by typical myopic macular degeneration (MMD), is the most detrimental subtype of high myopia and is of particular concern. However, little is yet known about its pathogenesis.

Recently, the relationship between pathologic myopia and inflammation has been brought to the fore, with increasing evidence for an inflammatory etiology of this disease (5, 6, 7). In our previous studies, the cytokine profiles of the aqueous humor of patients with pathologic myopia indicated an inflammatory intraocular microenvironment containing elevated pro-inflammatory monocyte chemotactic protein-1 and reduced anti-inflammatory interleukin-1 receptor antagonist (6). Moreover, upregulation of complement cascade was also found in the retina of guinea pigs with form deprivation-induced myopia that manifested retinal degeneration resembling MMD. These observations support a role of chronic inflammation in the pathogenesis of pathologic myopia. However, one crucial question remains unanswered: “Where does this inflammation start?”

Inflammation in many eye diseases, such as Graves’ disease, Sjögren’s syndrome, autoimmune retinopathy, and age-related macular degeneration (AMD), is secondary to a persistent autoimmune attack on ocular tissues (8, 9). In autoimmune retinopathy, circulating anti-retinal autoantibodies persistently attack the retinal cells, including Mϋller cells, bipolar cells, and photoreceptor cells, inducing apoptosis accompanied by cytokine release (8). In AMD, which involves chronic ocular inflammation, autoantibodies play vital roles in the dysfunction and death of retinal pigment epithelial (RPE) cells (8, 10, 11). Considering that MMD, the most typical and serious complication of pathologic myopia, involves the progressive loss of RPE cells within a pro-inflammatory microenvironment, we conjectured that autoimmunity might be involved in the pathogenesis of pathologic myopia. However, direct evidence remains absent.

Therefore, to test this hypothesis, we aim to explore the possible involvement of autoimmunity in pathologic myopia pathogenesis. An unbiased human proteome array (HuProt, CDI Laboratories, Inc) followed by a customized focused microarray was used to screen and verify high myopia-related autoantibodies in serum. We found that anti-LIMS1 autoantibody could distinguish high myopia from emmetropia, which was also associated with MMD grades. Further subgroup analysis revealed its actual relevance to pathologic myopia (MMD grades ≥2), rather than simple high myopia without typical MMD. Mechanistic investigation unveiled a detrimental effect of anti-LIMS1 autoantibody on RPE cells. These observations together support that serum anti-LIMS1 autoantibody could be used as a potential biomarker for pathologic myopia, which uncovers a previously unrecognized autoimmune etiology of MMD in pathologic myopia.

## Experimental Procedures

This study was approved by the Institutional Review Board of the Eye & Ear, Nose, and Throat (ENT) Hospital of Fudan University, Shanghai, China (no.2020068), and adhered to the tenets of the Declaration of Helsinki. Clinical data and serum samples were obtained from the Clinical Biological Sample Bank of the Shanghai High Myopia Study, in which personal information was masked and each participant was referred to by a consecutive code, in order to protect the confidentiality of human subjects. Written informed consents were obtained before participation from all subjects for the use of their clinical data and serum samples.

### Patients

We included consecutive patients who attended the Eye & ENT Hospital of Fudan University between August 2020 and December 2021. Subjects, whose axial lengths (ALs) obtained with IOLMaster 700 (Carl Zeiss AG) of bilateral eyes were between 22 and 24.5 mm, were included in the emmetropia group, and subjects with ALs of bilateral eyes longer than 26 mm were included in the high myopia group. Exclusion criteria were (1) other ocular diseases, such as glaucoma, uveitis, AMD, and so on.; (2)previous ocular trauma or surgery; (3) systemic autoimmune diseases, such as systemic lupus erythematosus, inflammatory bowel disease, diabetes mellitus, and so on; (4) a history of tumor; (5) and no clear fundus photograph. We also additionally included 20 patients diagnosed with AMD to ascertain the specificity of the biomarker.

The MMD of the highly myopic eyes was graded from 0 to four based on the fundus photograph acquired with a 45-degree digital retinal camera (Canon Inc), according to the International Meta-Analysis for Pathologic Myopia (META-PM) Classification System, an international photographic classification system proposed by the META-PM study group (12). Highly myopic subjects were divided into different groups based on the MMD grade of the worse eye. Highly myopic patients, whose both eyes were classified as MMD grade 0 to 1 (no myopic retinal degenerative lesion and tessellated fundus) were included into the simple high myopia subgroup, and those with either eye of MMD grade 2 to 4 (diffuse chorioretinal atrophy, patchy chorioretinal atrophy, and macular atrophy) were included into the pathologic myopia subgroup (12, 13, 14).

### Serum Sample Collection and Storage

Respectively 1 ml venous blood per subject for array profiling and ELISA, and 10 ml venous blood per subject for IgG purification were collected into coagulation-promoting vacuum blood tubes. Blood samples were centrifuged at 3000 rpm for 10 min at 4 °C within 4 h. The top serum layer was aspirated and stored at −80 °C until use.

### Phase I: Serum Profiling with HuProt Arrays

In phase I, the serum samples were probed with HuProt v3.1 arrays (CDI Laboratories, Inc), which comprised more than 20,000 unique human full-length proteins, according to the manufacturer’s instructions. Briefly, the HuProt array is based on the sandwich immunoassay principle. More than 20,000 unique human proteins are immobilized in duplicate in specific locations on the surface of the array membrane. After blocking, the HuProt array was incubated in 3 ml of serum sample, diluted 1:1,000, to capture the autoantibodies corresponding to each protein spot. It was then incubated with Cy3-conjugated goat anti-human IgG (Jackson, 109-165-008) and Alexa Fluor 647-affinipure donkey anti-human IgM (Jackson, 709-605-073), both diluted 1:2,000, to fluorescently label the IgG and IgM forms of the captured autoantibodies, respectively. Finally, the HuProt assays were scanned with the GenePix 4000B Microarray Scanner (Molecular Devices) and analyzed with the GenePix Pro 6.0 software (Molecular Devices).

The signal intensity of each protein spot was calculated as the ratio of the foreground to background median signal intensity. Each protein was duplicated in pairs, thus, the signal intensity of each autoantibody was calculated as the mean value of the two protein spots. The normalized signal intensity was calculated as previously reported ([Rp − ¯N]/SD, where Rp is the signal intensity of the autoantibody, ¯N and SD are the mean value and standard deviation of signal intensity of all autoantibodies on the microarray, respectively) to avoid noise disturbance (15, 16). To screen for biomarkers with enhanced specificity, a stringent cut-off (normalized signal value ≥15) was used to identify the positives.

High myopia-related candidate autoantibodies were identified with the following criteria: (1) positive rate (proportion of samples in which the autoantibody was positive) in the high myopia group > positive rate in the emmetropia group; (2) a positive rate in the emmetropia group of <30%; and (3) *p* value of <0.05 when the positive rate of two groups were compared.

### Phase II: Construction of Focused Microarrays and Serum Assay

In phase II, we customized focused microarrays (CDI Laboratories, Inc), which included all the candidate autoantibodies screened from HuProt arrays. Every OPEpoxy Slide contained 12 duplicated sub-arrays, and a 12-hole rubber gasket divided each microarray into individual chambers, to perform 12 serum assays simultaneously. The protocol of the focused microarray was similar to that of the HuProt array, except that 200 μl of serum, diluted 1:100, was incubated in each chamber.

Because there were 36 different comparisons, the autoantibody was identified as a high myopia-related autoantibody when (1) the fold change in signal intensity (mean signal intensity of high myopia group/mean signal intensity of emmetropia group) was >1.3; (2) the *p* value of Student’s *t* test compared between emmetropia and high myopia groups after Bonferroni correction was <0.00139 (0.05/36); (3) the area under the receiver operating characteristic (ROC) curve (AUC) for distinguishing high myopia from emmetropia was >0.6.

### Immunofluorescent Staining

For immunofluorescent staining, tissue from the posterior pole of an eye (from a 76-year-old human male donor at the Eye & ENT Hospital of Fudan University) and human RPE cells (ARPE-19) were fixed with 4% paraformaldehyde, permeabilized with (phosphate-buffered saline) PBS containing 0.3% Triton X-100 and blocked with 3% bovine serum albumin. The cells were probed with a primary antibody overnight and then with a secondary antibody. Finally, nuclei were stained with 4′,6-diamidino-2-phenylindole (DAPI). Images were taken with a confocal microscope (Leica Microsystems). To label the LIMS1 antigen, the anti-LIMS1 antibody (LSBio, LS-C169391) was used as the primary antibody, followed by an Alexa Fluor 488-conjugated anti-mouse secondary antibody (Beyotime, A0428). The cytoskeleton was visualized by labeling vinculin with an anti-vinculin antibody (Abcam, ab129002), followed by an Alexa Fluor 488-conjugated goat anti-rabbit secondary antibody (Beyotime, A0423). F-actin was visualized with rhodamine-conjugated phalloidin (Beyotime, C2207S). An anti-intracellular adhesion molecule 1 (ICAM-1) antibody (Servicebio, GB11106) and an anti-occludin antibody (Servicebio, GB111401) were used as primary antibodies to label ICAM-1 and occluding, respectively, followed by an Alexa Fluor 488-conjugated goat anti-rabbit antibody (Beyotime, A0423) as the secondary antibody.

### *In situ* Hybridization

For the *in situ* hybridization study of the human eye (from a 76-year-old human male donor at the Eye & ENT Hospital of Fudan University), RNASweAMITM (Servicebio, GF001) was used to hybridize with the ocular mRNA *in situ*, to determine the expression of the LIMS1 antigen according to the manufacturer’s protocol.

### Western Blotting Analysis

All proteins of the human lens epithelial cell line (SRA01/04), the human trabecular meshwork cell line (HTMC), and the human RPE cell line (ARPE-19) were extracted with RIPA lysis buffer. The protein extracts (20 μg per lane) were separated by sodium dodecyl sulfate-polyacrylamide gel electrophoresis (Biorad) and electrotransferred onto a polyvinylidene difluoride membranes (Amersham). After blocking, the membrane was incubated with the primary anti-LIMS1 antibody (LSBio, LS-C169391), followed by a horseradish peroxidase (HRP)-conjugated goat anti-mouse IgG (Beyotime, A0216). Finally, the proteins were visualized with the ECL Plus Western Blotting Substrate (Biosharp, BL520A).

### Phase III: Antibody Typing with Enzyme-linked Immunosorbent Assays (ELISAs)

Individual wells of Grenier 96-well plates were coated with 50 ng of LIMS1 protein overnight. To perform the standard ELISAs, the plate was incubated independently with 1:100-diluted serum samples and an antibody directed against each type (IgG, IgM, IgA, or Ig E) or subtype (IgG1-4) of immunoglobulin (goat anti-human IgG-HRP [Jackson, 109-035-003] to label IgG, goat anti-human IgM-HRP [Abcam, ab97205] to label IgM, rabbit anti-human IgA-HRP [Abcam, ab8510] to label IgA, goat anti-human IgE-HRP [Thermo Fisher, A18793] to label IgE, mouse anti-human IgG1-HRP [Thermo Fisher, A-10648] to label IgG1, mouse anti-human IgG2-HRP [Thermo Fisher, MH1722] to label IgG2, mouse anti-human IgG3-HRP [Thermo Fisher, 05-3620] to label IgG3, mouse anti-human IgG4-HRP [Thermo Fisher,A-10654] to label IgG4) diluted according to the manufacturer’s instructions. The immunoreactivity signals were read as the absorbance at wavelength of 450 nm (A_450_). For normalization, the antibody levels of the individual serum samples were measured as a ratio to the mean A_450_ value of emmetropia group.

### Purified IgG from Serum Sample

The IgG from the serum samples of subjects with emmetropia, simple high myopia, or pathologic myopia was purified, respectively (each type of IgG was purified from the serum samples from six subjects in each group), by affinity chromatography using rProtein L Beads (4FF, Smart-Lifesciences, Changzhou, China), and designated as “emmetropia IgG,” “simple HM IgG,” or “PM IgG,” respectively. Half of PM IgG was then passed through an LIMS1-antigen-conjugated affinity column to remove any LIMS1-specific antibody, yielding anti-LIMS1 depleted PM IgG. The purified IgG was stored at −80 °C until use.

### ARPE-19 Cell Culture and Treatment with Purified IgG

ARPE-19 cells were placed in a culture dish and incubated in F-12 medium (Thermo Fisher Scientific, C11330500BT) supplemented with 10% fetal bovine serum and grown at 37 °C under 5% CO_2_. The medium was replaced every other day. After treatment with purified IgG, the ARPE-19 cells were seeded in a 96-well plate at 8 × 10^4^ cells/ml, and incubated in serum-free F-12 medium containing purified IgG (final concentration 500 μg/ml) for 24 h at 37 °C for the analysis of the cytoskeleton, tight junction, ICAM-1, and cytokine.

### Transepithelial Electrical Resistance (TEER) Study

ARPE-19 cells were seeded in the upper compartment of collagen-coated Transwell tissue culture inserts (0.4 μm pore size, effective growth area 0.33 cm^2^, 1.8 × 10^4^ cells/insert; Costar, 3413) and grown to confluence. They were then incubated with serum-free F-12 medium containing purified IgG (final concentration 500 μg/ml) for 24 h at 37 °C. The TEER values of the cell layers were measured with a Millicell electrical resistance apparatus (Endohm-6 EVOM, World Precision Instruments) and normalized to the mean value of the emmetropia group (17).

### Paracellular Permeability to 10-kDa Dextran

Monolayer RPE cells were placed on 24-well collagen-coated Transwell tissue culture inserts (0.4 μm pore size; Costar, 3413), and incubated in a medium containing the specific purified IgG (final concentration 500 μg/ml) for 24 h at 37 °C. We then replaced the medium and added fluorescein isothiocyanate (FITC)-conjugated dextran fluorescence (10 kDa; Aladdin, F291043) into the luminal insert (final concentration 1 mg/ml). After 1 h, we collected 50 μl of medium from the bottom and abluminal chambers, and then transferred the samples into 96-well black plates. The fluorescent signals were read at A_490_/E_520_ with a SpectraMax M2 microplate reader (Molecular Devices), and the ratio of the signal intensity in the bottom chamber to that in the abluminal chamber reflected the paracellular permeability of the cells. This value was then normalized to the mean value of the emmetropia group for statistical analysis.

### Cytokine Screening With a Multiplexed Fluorescent Bead-Based Immunoassay

The culture supernatants of ARPE-19 cells treated with serum-free F-12 medium containing purified IgG (final concentration 500 μg/ml) for 24 h at 37 °C were collected and assayed for 48 cytokines with the Bio-Plex Pro Human Cytokine Assay (Bio-Rad, 12007283). Quintuplicate cultures were established and their supernatants were detected. The fluorescent signals were detected with the Luminex 200 detector and converted to corresponding concentrations (pg/ml) based on the standard curves provided with the Bio-Plex Manager software (Luminex Corporation).

### Experimental Design and Statistical Rationale

We conducted a three-phase study to identify serological autoantibodies specific for high myopia (Supplemental Table S1 and Supplemental Fig. S1). In phase I, high myopia-related candidate autoantibodies were unbiasedly screened with HuProt arrays in a small sample of patients, including 26 with high myopia and 10 with emmetropia. In phase II, the candidates autoantibodies identified in phase I were validated in a larger cohort, including 222 patients with high myopia and 130 with emmetropia. The correlation between the high myopia-related autoantibody and pathologic myopia was investigated. In phase III, the antibody classes (IgG, IgM, IgA, and IgE) and subclasses (IgG1-4) of the identified autoantibody were determined using ELISAs of serum samples from an independent cohort consisting of 20 patients with pathologic myopia, 20 with simple high myopia, 20 with emmetropia, and 20 with AMD.

All continuous data are expressed as means ± standard deviation and categorical data are expressed as frequencies and percentages for each category. For continuous data, two groups were compared with Student’s *t* test, and more than two groups were compared with one-way analysis of variance (ANOVA) and the least significant difference (LSD) post hoc test. Categorical data were compared with a χ2 test or Fisher’s exact test (positive rate in phase I). The relationships between continuous variables were assessed using Pearson’s correlation coefficient. A ROC analysis was performed to identify the AUC, optimal cut-off value, sensitivity, and specificity. The optimal cut-off value for each autoantibody was identified by specificity ≥90% and the highest Youden index (sensitivity + specificity −1). A multivariate binomial logistic regression analysis was performed to determine whether the identified autoantibody was significantly associated with pathologic myopia. During the analysis of cytokines, because there were 48 different comparisons, *p* values of <0.00104 (0.05/48) after Bonferroni correction were considered statistically significant. *p* values <0.05 were considered statistically significant for all other analyses. All analyses were performed with using IBM SPSS v22.0 (Chicago).

## Results

### Phase I: 18 High Myopia-related Candidate Autoantibodies were Identified Using HuProt Arrays

**Fig. 1:**
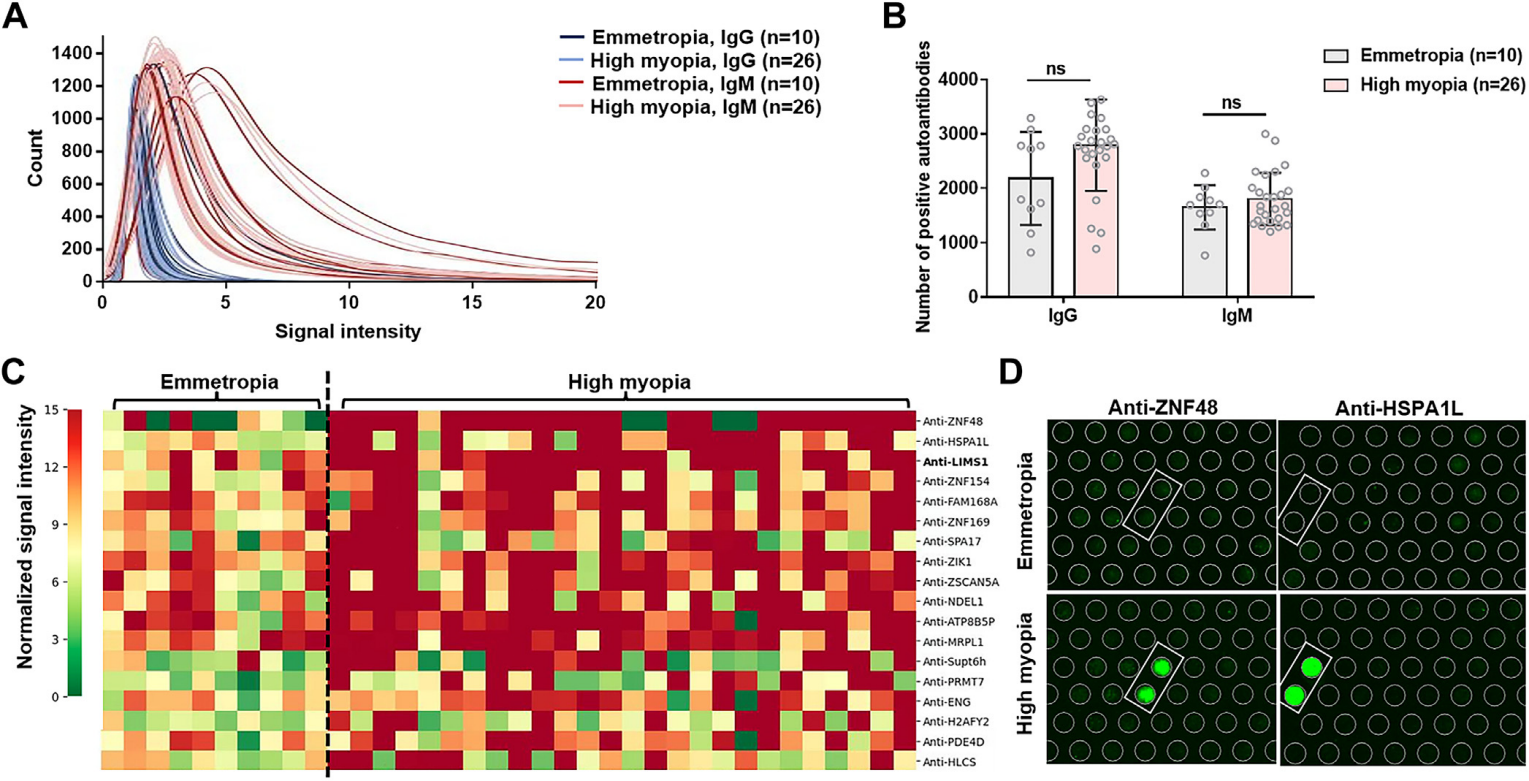
**Phase I: 18 highmyopia-related candidate autoantibodies were identified with HuProt arrays.***A*, distribution curves of signal intensities of all autoantibodies detected with the HuProt arrays. *B*, numbers of positive autoantibodies detected with each HuProt array (IgG *p* = 0.060, IgM *p* = 0.370). *C*, heatmap of the normalized signal intensities of 18 high myopia-related candidate autoantibodies identified with HuProt arrays (all *p* < 0.05). *D*, representative images of fluorescent signals of the two most-significant candidate autoantibodies in serum samples from patients with high myopia or emmetropia, detected with HuProt arrays. Results are expressed as mean ± SD. N = 10 for the emmetropia group and N = 26 for the high myopia group (*A*–*C*). The significance of differences was determined with two-sided Student’s *t* test (*B*), and Fisher’s exact test (*C*). No significant difference is represented as ns.

In the screening stage, the signal intensity distribution showed that the majority of tested autoantibodies were not detected in these serum samples (Fig. 1*A*). The number of positive autoantibodies identified in each sample ranged from approximately 800 to more than 3600, regardless of the origin of the samples (Student’s *t* test, both *p* > 0.05; Fig. 1*B*).

With the comparisons of positive rates of each autoantibody between emmetropia and high myopia groups, a total of 18 autoantibodies, including anti-ZNF48, anti-HSPA1L, etc. were identified as high myopia-related candidate autoantibodies (Fisher’s exact test, all *p* < 0.05; Fig. 1*C*, Supplemental Table S2). Figure 1*D* illustrates representative fluorescent signals of the two most-significant candidate autoantibodies.

### Phase II: Anti-LIMS1 was Validated as a High Myopia-Related Autoantibody With Focused Microarrays

**Fig. 2:**
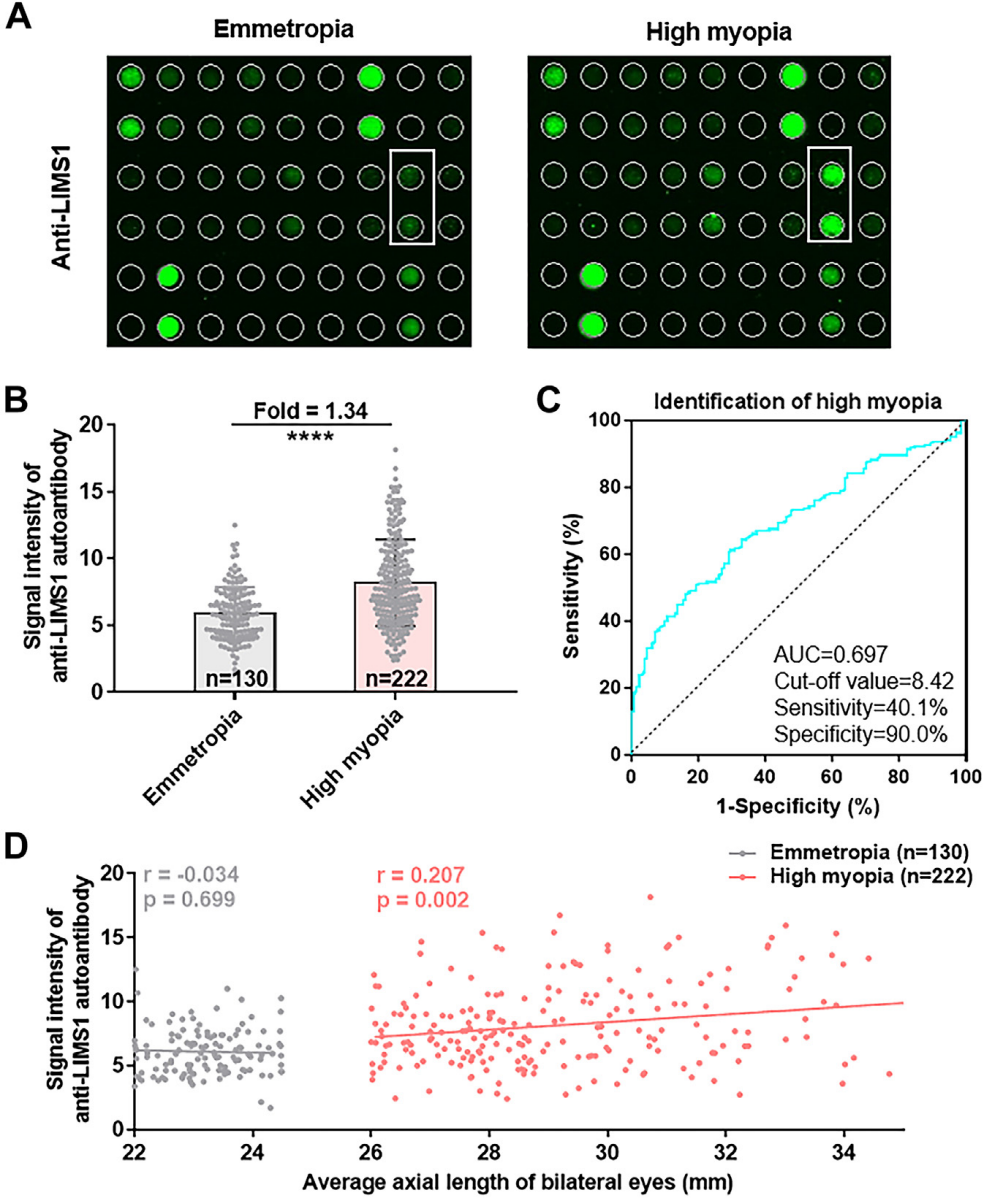
**Phase II: Anti-LIMS1 autoantibody was validated as a high myopia-related autoantibody using customized focused microarrays.***A*, representative image of fluorescent signals of anti-LIMS1 autoantibody in serum samples from patients with emmetropia and high myopia, detected with focused microarrays. *B*, comparison of the signal intensities of anti-LIMS1 autoantibody in patients with emmetropia or high myopia (*p* = 6.97e-10). *C*, receiver–operating characteristic analysis of the performance of the signal intensity of anti-LIMS1 autoantibody for identifying high myopia. *D*, correlations between the average axial length of bilateral eyes and the signal intensity of anti-LIMS1 autoantibody in emmetropia and high myopia groups. Results are expressed as means ± SD. N = 130 for the emmetropia group and N = 222 for the high myopia group (*B*–*D*). The significance of differences was determined with two-sided Student’s *t* test (*B*). Associations between variables were evaluated using two-sided Pearson’s correlation coefficient tests (*D*). ∗∗∗∗ *p* < 0.0001.

Using a focused microarray, the anti-LIMS1 autoantibody was further verified as the most-significant autoantibody distinguishing highly myopic subjects from emmetropic controls (fold change in signal intensity = 1.34, Student’s *t* test with Bonferroni correction, *p* = 6.97e-10; Fig. 2, *A* and *B*, Supplemental Table S3). The ROC analysis showed that, when the signal intensity of anti-LIMS1 autoantibody was used to identify high myopia, the AUC was 0.697 and the cut-off value was 8.42, with 40.1% sensitivity and 90.0% specificity (Fig. 2*C*).

Pearson’s correlation analysis showed a positive correlation between the average AL of bilateral eyes and the signal intensity of the anti-LIMS1 autoantibody in highly myopic subjects (r = 0.207, *p* = 0.002; Fig. 2*D*), but not in the emmetropic controls (*p* > 0.05; Fig. 2*D*).

### Anti-LIMS1 Autoantibody is Involved in Pathologic Myopia but Not in Simple High Myopia

**Fig. 3:**
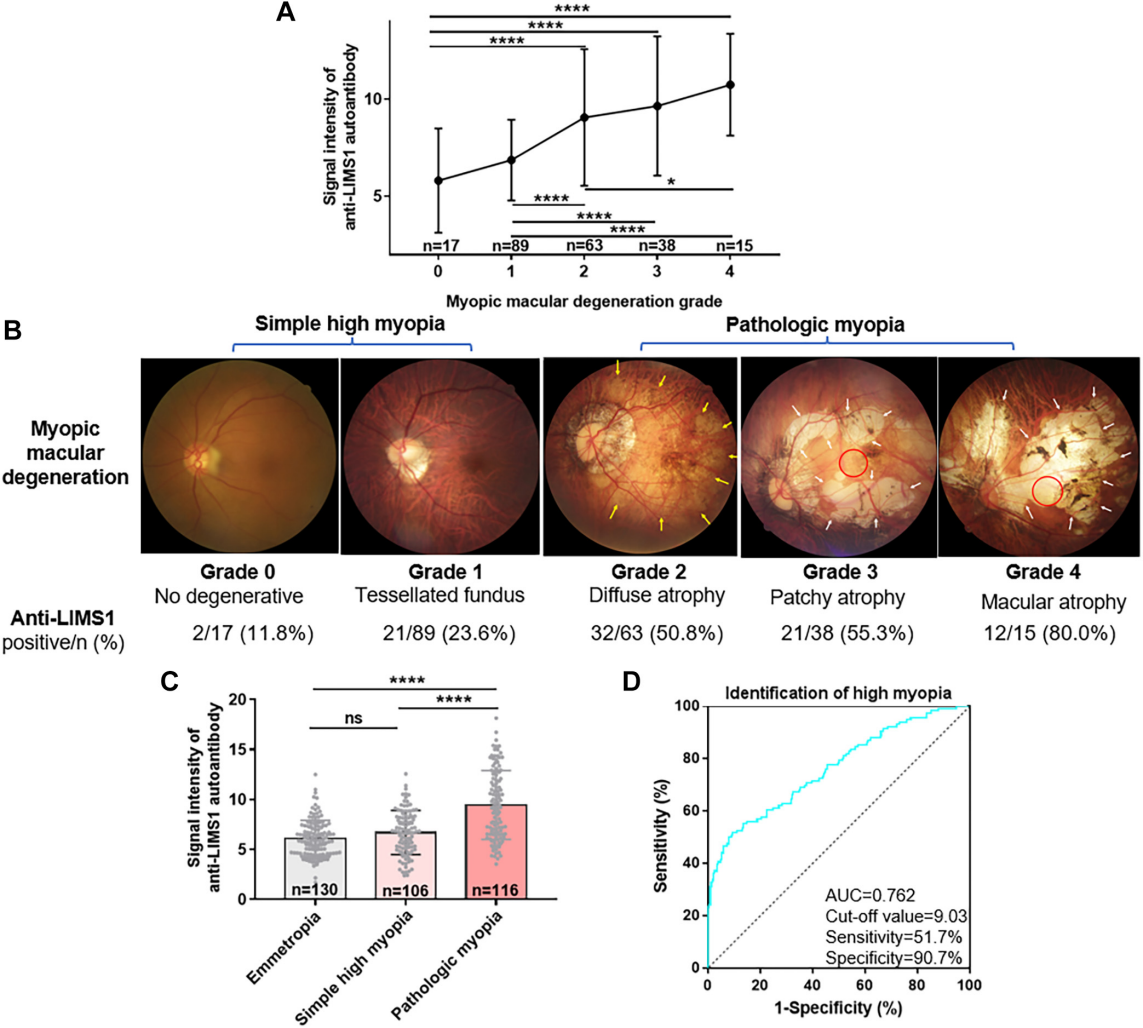
**Subgroup analysis revealed relevance of anti-LIMS1 autoantibody to pathologic myopia rather than simple high myopia.***A*, signal intensity of anti-LIMS1 autoantibody in patients with different grades of myopic macular degeneration (MMD) (*p* = 2.91e-10). *B*, positive rates of anti-LIMS1 autoantibody in patients with different MMD grades (*p* = 0.000002). A signal intensity of the anti-LIMS1 autoantibody exceeded the cut-off value for identifying high myopia (8.42) was considered a positive hit. *Yellow* and *white arrows* indicate area of diffuse and patchy chorioretinal atrophy, respectively, and *red circles* indicate the foveal region. *C*, comparison of the signal intensity of anti-LIMS1 autoantibody among emmetropia, simple high myopia (0–1 MMD grade) and pathologic myopia (MMD grade ≥2) groups (*p* = 1.45e-22). *D*, receiver–operating characteristic analysis of the performance of the signal intensity of anti-LIMS1 autoantibody for identifying pathologic myopia (MMD grade ≥2). Results are expressed as means ± SD (continuous data) or frequencies and percentages for each grade (categorical data). N = 17 for grade 0, N = 89 for grade 1, N = 63 for grade 2, N = 38 for grade 3, N= 15 for grade 4 (*A* and *B*). N= 130 for the emmetropia group, N = 106 for the simple high myopia group, and N = 116 for the pathologic myopia group (*C* and *D*). The significance of differences was determined using one-way ANOVA and the LSD post hoc test (*A* and *C*), or the χ2 test (*B*). ∗∗∗∗ *p* < 0.0001, ∗ *p* < 0.05, and ns represents no significant difference.

In addition to the correlation between the anti-LIMS1 autoantibody and AL, we investigated its potential relationship with MMD, a typical complication of pathologic myopia. The signal intensity of anti-LIMS1 autoantibody increased significantly with the grade of MMD according to the META-PM Classification System (ANOVA, *p* < 0.0001; Fig. 3*A*). Meanwhile, the positive rate of anti-LIMS1 autoantibody also showed a similar trend (χ2 test, *p* < 0.0001; Fig. 3*B*).

We also conducted a subgroup analysis, dividing the highly myopic patients into those with simple high myopia or pathologic myopia based on the META-PM Classification System (simple high myopia: high myopia with 0–1 MMD grade; pathologic myopia: high myopia with MMD grade ≥2) (18). The mean signal intensity of anti-LIMS1 autoantibody was greatest in the pathologic myopia group (ANOVA with LSD post hoc test, both *p* < 0.0001; Fig. 3*C*), but did not differ significantly between the emmetropia and simple high myopia groups (ANOVA with LSD post hoc test, *p* > 0.05; Fig. 3*C*). When the signal intensity of anti-LIMS1 autoantibody was used to identify pathologic myopia, the ROC analysis showed that the AUC was 0.762, with a cut-off value of 9.03, reaching 51.7% sensitivity and 90.7% specificity (Fig. 3*D*). Multiple binomial logistic regression, including age, sex, average AL of bilateral eyes, and THE signal intensity of anti-LIMS1 autoantibody, showed that average AL of bilateral eyes (β = 1.878, 95% confidence interval [CI] 1.612–2.188, *p* < 0.001) and the signal intensity of anti-LIMS1 autoantibody (β = 1.448, 95%CI 1.267–1.656, *p* < 0.001) were significantly and independently associated with pathologic myopia. Therefore, the anti-LIMS1 autoantibody is likely to be related to pathologic myopia-related autoantibody.

### LIMS1 Antigen is Expressed in RPE Cells

**Fig. 4:**
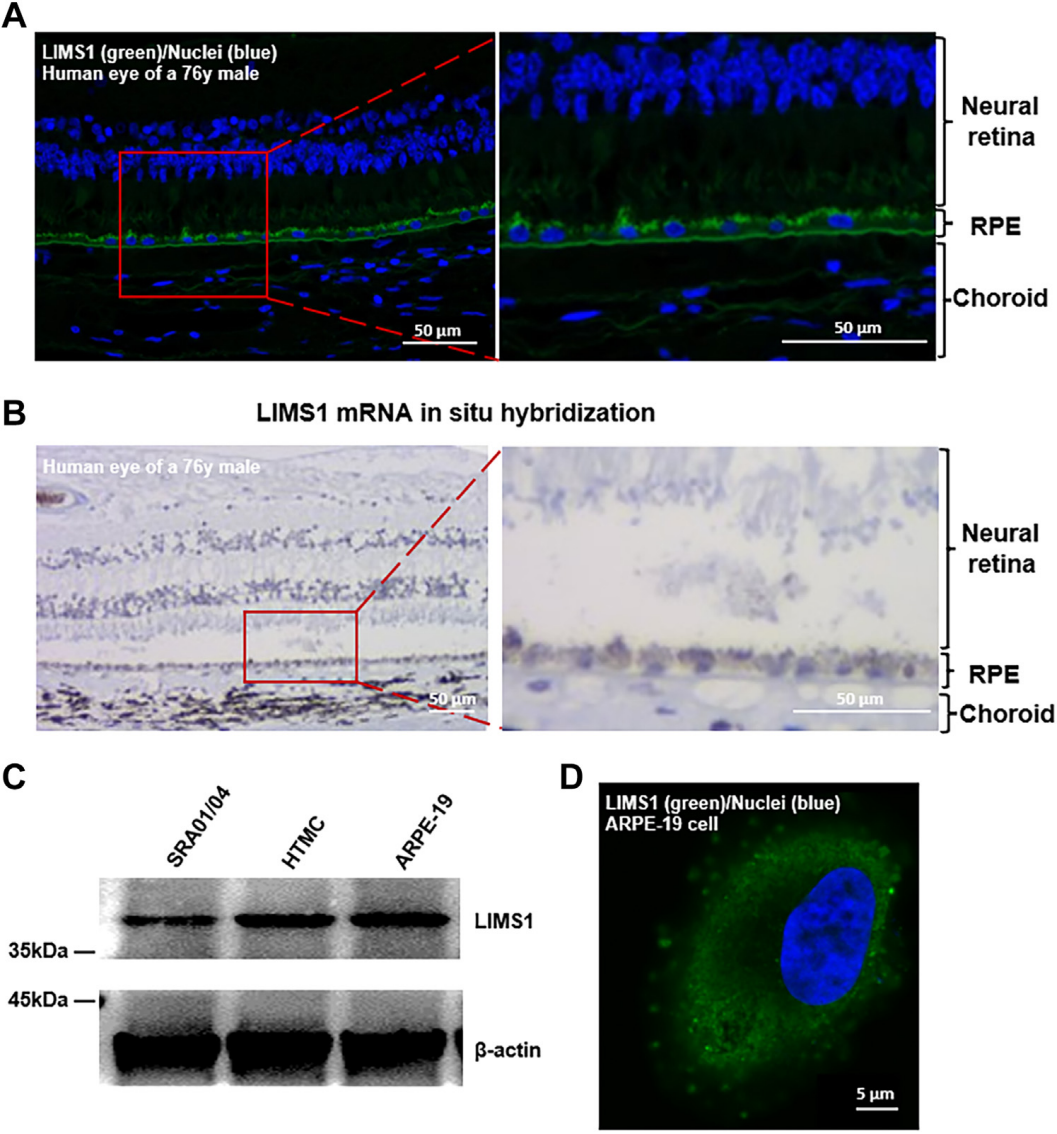
**LIMS1 antigen is expressed in human retinal pigment epithelium (RPE) cells.***A*, immunofluorescent image of LIMS1 staining in the posterior pole of a human eye showing high expression in RPE cells. Scale bar: 50 μm. *B*, LIMS1 mRNAs *in situ* hybridization in the posterior pole of a human eye showing high expression in RPE cells. Scale bar: 50 μm. *C*, Western blotting of LIMS1 expression in a human lens epithelial cell line (SRA01/04), a human trabecular meshwork cell line (HTMC), and a human RPE cell line (ARPE-19). *D*, immunofluorescent image of LIMS1 staining in ARPE-19 cells. Scale bar: 5 μm.

After the identification of the circulating anti-LIMS1 autoantibody as a pathologic myopia-related autoantibody, we investigated whether the LIMS1 antigen was expressed in the human eye. Immunofluorescent staining showed the presence of LIMS1 in the posterior pole of the human eye, mainly in the RPE (Fig. 4*A*), a single cell layer between the neurosensory retina and the choroid, forming the outer blood-retinal barrier (oBRB). *In situ* hybridization of mRNA also showed intense LIMS1 expression in the RPE (Fig. 4*B*). The expression of LIMS1 in several human ocular cell lines was also confirmed with western blotting (Fig. 4*C*), with the highest expression in RPE cells (ARPE-19). Immunofluorescent staining revealed the clear expression of LIMS1 in ARPE-19 cells (Fig. 4*D* and Supplemental Fig. S2).

### Phase III: Anti-LIMS1 Autoantibody in Pathologic Myopia Belongs to IgG1/IgG2/IgG3 Subclasses

**Fig. 5:**
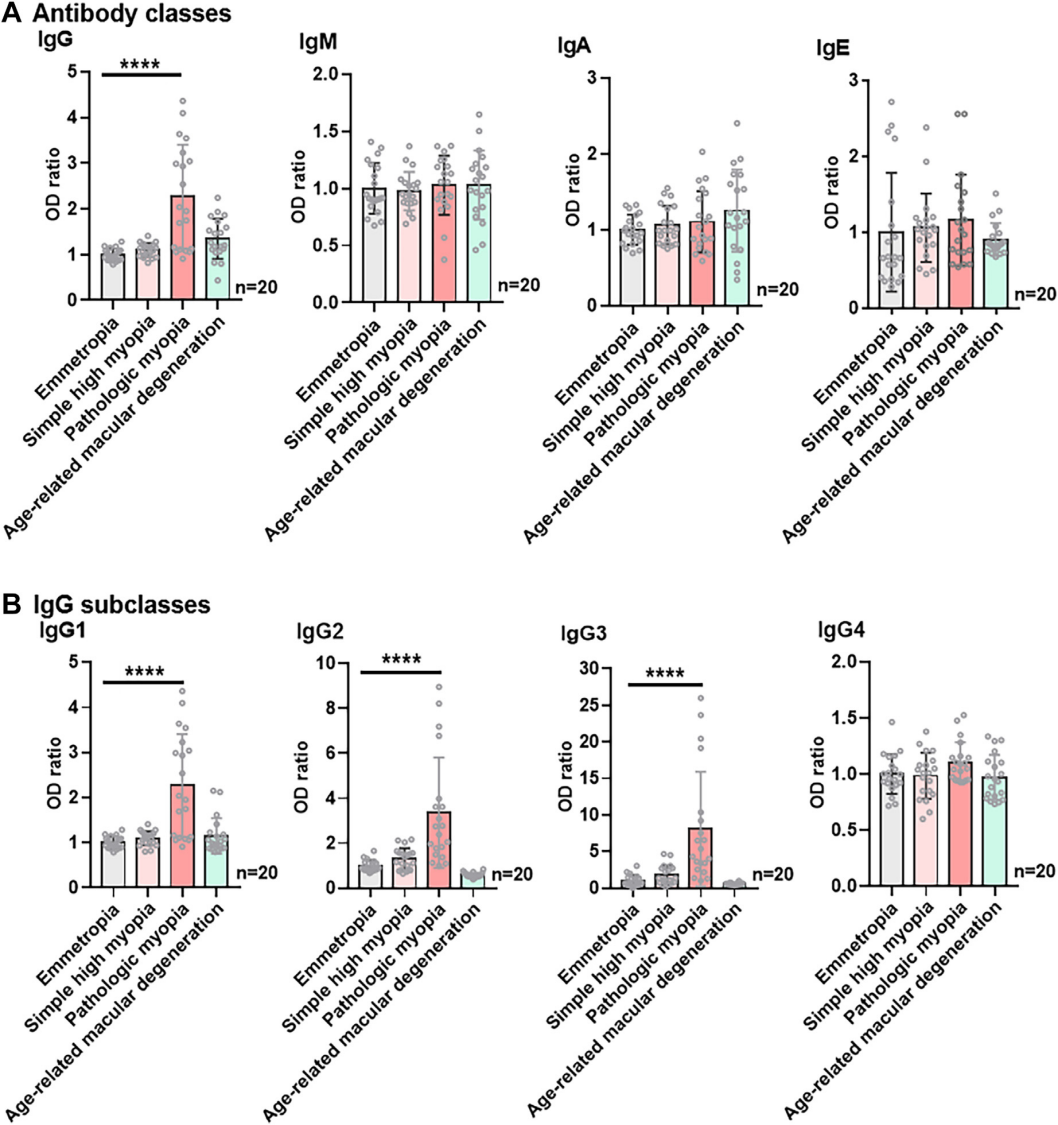
**Phase III: Anti-LIMS1 autoantibody in pathologic myopia predominantly belonged to IgG1/IgG2/IgG3 subclasses.***A*, examination of the levels of different anti-LIMS1 autoantibody classes in serum sample by ELISA (n = 20/group, IgG *p* = 1.06e-8, IgM *p* = 0.890, IgA *p* = 0.184, IgE *p* = 0.528). *B*, examination of the serum levels of anti-LIMS1 autoantibody of different IgG subclasses by ELISA (n = 20/group, IgG1 *p* = 2.75e-9, IgG2 *p* = 2.47e-9, IgG3 *p* = 4.88e-8, IgG4 *p* = 0.147). Results are expressed as mean ± SD. OD value was normalized to loading the mean value of emmetropia group as a ratio for statistical analysis. The significance of differences was determined using one-way ANOVA with the LSD post hoc test. ∗∗∗∗ *p* < 0.0001, and ns represents no significant difference.

We further explored the antibody classes and subclasses of the anti-LIMS1 autoantibody in pathologic myopia. As shown in Figure 5*A*, only the serum level of the IgG class of anti-LIMS1 autoantibody was elevated in patients with pathologic myopia (ANOVA, *p* < 0.0001), and no significant differences were detected in the IgM, IgA, and IgE classes among the groups (ANOVA, all *p* > 0.05). The IgG subclasses of the anti-LIMS1 autoantibody in pathologic myopia were predominantly IgG1/IgG2/IgG3 (ANOVA, all *p* < 0.0001; Fig. 5*B*).

### Anti-LIMS1 Autoantibody Disrupts the Barrier Function of the RPE

Given the high expression of the LIMS1 antigen in RPE cells and the IgG dominance of anti-LIMS1 autoantibody, we treated ARPE-19 cells with purified IgG from the sera of subjects with emmetropia (emmetropia IgG), simple high myopia (simple HM IgG), or pathologic myopia (PM IgG), or with PM IgG depleted of anti-LIMS1 autoantibody (anti-LIMS1 depleted PM IgG), respectively, to examine its effects on RPE cells.

**Fig. 6:**
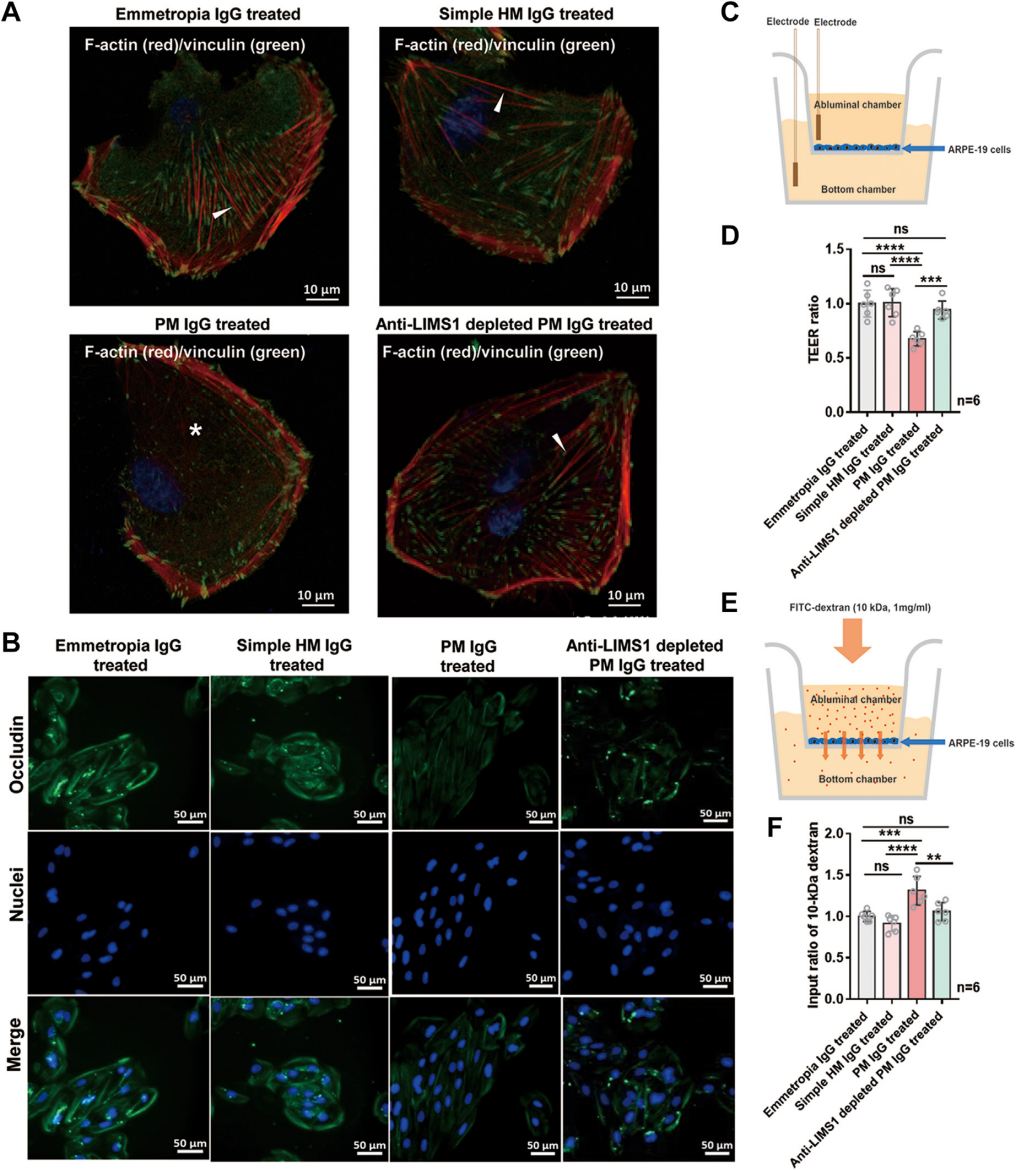
**Anti-LIMS1 autoantibody disrupts the barrier function of ARPE-19 cells.***A*, immunofluorescent images of F-actin (*red*) and vinculin (*green*) staining in the ARPE-19 cells after exposure to purified IgG (500 μg/ml) extracted from the serum samples of subjects with emmetropia (emmetropia IgG), simple high myopia (simple HM IgG), pathologic myopia (PM IgG), or PM IgG depleted of anti-LIMS1 autoantibody (anti-LIMS1 depleted PM IgG). Scale bar: 10 μm. *White arrows* indicate radially arranged actin bundles, and the white asterisk indicates abnormal scattering of F-actin. *B*, immunofluorescent images of occludin staining in ARPE-19 cells treated with each purified IgG (500 μg/ml). Scale bar: 50 μm. *C*, experimental setup of transepithelial electrical resistance (TEER) measurements. ARPE-19 cells were seeded on the abluminal chamber. Two chopstick electrodes of the Millicell electrical resistance apparatus were placed in the abluminal and bottom chambers, and the TEER value of the cell layer was measured. *D*, mean TEER values of ARPE-19 cell layers after exposure to each purified IgG (500 μg/ml, N = 6/group, *p* = 0.000046). *E*, experimental setup to measure paracellular permeability to 10-kDa dextran. ARPE-19 cells were seeded in the abluminal chamber and then added fluorescein isothiocyanate (FITC)-dextran fluorescence into the abluminal chamber (10 kDa, 1 mg/ml). After 1 h, the ratio of the fluorescence signal intensity in the bottom chamber to that in the abluminal chamber was determined as an indicator of paracellular permeability. *F*, permeability of the ARPE-19 cell layer to 10-kDa dextran after exposure to each purified IgG (500 μg/ml, N = 6/group, *p* = 0.000069). Results are expressed as means ± SD. TEER values and input of dextran were normalized to the loading mean value for emmetropia as a ratio for statistical analysis (*D* and *F*).The significance of differences was determined using one-way ANOVA with the LSD post hoc test (*D* and *F*). ∗∗∗∗ *p* < 0.0001, ∗∗∗ *p* < 0.001, ∗∗ *p* < 0.01, and ns represents no significant difference.

The proper formation of the cytoskeleton and the presence of tight junctions are critical factors for maintaining the barrier function of the RPE. Therefore, we examined the cytoskeletal organization of RPE cells by co-staining them for F-actin and vinculin. After 24 h, the ARPE-19 cells exposed to PM IgG showed obvious down-regulation of F-actin and vinculin, disorganization of the actin bundles, and resulting in wrinkling of the cell body, as observed in confocal images. These effects were attenuated after depleting the anti-LIMS1 autoantibody (Fig. 6*A* and Supplemental Fig. S3). ARPE-19 cells treated with PM IgG also showed obvious down-regulation of occludin, a tight junction protein abundantly expressed on RPE cells, compared with those treated with emmetropia IgG or simple HM IgG. This effect was also attenuated after depleting the anti-LIMS1 autoantibody (Fig. 6*B* and Supplemental Fig. S3).

To determine whether the structural changes in the RPE cell cytoskeleton and tight junctions lead to the disruption of the barrier function of the oBRB, we further investigated the changes in the TEER value and the permeability of oBRB to 10-kDa dextran after exposure to each purified IgG. After 24-h exposure to PM IgG, the TEER value decreased (ANOVA with LSD post hoc test, both *p* < 0.0001; Fig. 6, *C* and *D*) and the permeability of oBRB to 10-kDa dextran increased (ANOVA with LSD post hoc test, both *p* < 0.001; Fig. 6, *E* and *F*) compared with those treated with emmetropia IgG or simple HM IgG, but not after exposure to anti-LIMS1 depleted PM IgG (ANOVA with LSD post hoc test, all *p* > 0.05, Fig. 6, *D* and *F*). These findings demonstrate that the detrimental effects of the anti-LIMS1 autoantibody on the cytoskeleton and tight junctions may disrupt the barrier function of RPE in patients with pathologic myopia.

### Anti-LIMS1 Autoantibody Induces a Pro-Inflammatory State in RPE Cells

A pro-inflammatory microenvironment has previously been demonstrated in eyes with pathologic myopia. To determine whether the anti-LIMS1 autoantibody is associated with the pro-inflammatory intraocular microenvironment in pathologic myopia, we detected the expression of ICAM-1 in RPE cells and the levels of cytokines in the supernatants of ARPE-19 cells after 24-h exposure to emmetropia IgG, simple HM IgG, PM IgG, or anti-LIMS1 depleted PM IgG.

**Fig. 7:**
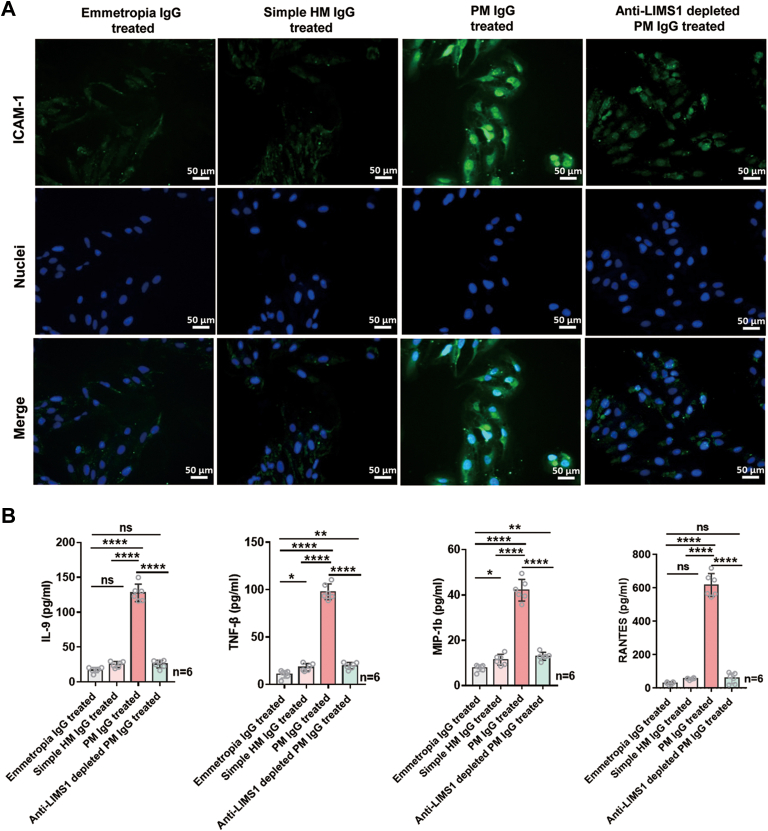
**Anti-LIMS1 autoantibody induces a pro-inflammatory state in ARPE-19 cells.***A*, immunofluorescent images of ICAM-1 staining in ARPE cells after exposure to purified IgG (500 μg/ml) extracted from the serum samples of subjects with emmetropia (emmetropia IgG), simple high myopia (simple HM IgG), pathologic myopia (PM IgG), or PM IgG depleted of anti-LIMS1 autoantibody (anti-LIMS1 depleted PM IgG). Scale bar: 50 μm. *B*, concentrations of cytokines in the culture supernatants of ARPE-19 cells treated with emmetropia IgG, simple HM IgG, PM IgG, or anti-LIMS1 depleted PM IgG (500 μg/ml) detected by a multiplexed fluorescent bead-based immunoassay (N = 6/group, IL-9 *p* = 7.26e-17, TNF-β *p* = 1.47e-17, MIP-1b *p* = 1.26e-14, RANTES *p* = 3.22e-17). Results are expressed as means ± SD. The significance of differences was determined using one-way ANOVA with the LSD post hoc test (*B*). ∗∗∗∗ *p* < 0.0001, ∗∗ *p* < 0.01, ∗ *p* < 0.05, and ns represents no significant difference.

ICAM-1 staining in ARPE-19 cells showed that ICAM-1 was negligibly expressed in the ARPE-19 cells treated with emmetropia IgG or simple HM IgG (Fig. 7*A* and Supplemental Fig. S3). However, the expression of ICAM-1 increased substantially in the ARPE-19 cells after exposure to PM IgG, and this effect was attenuated after the removal of the anti-LIMS1 autoantibody (Fig. 7*A* and Supplemental Fig. S3).

We used a multiplexed fluorescent bead-based immunoassay to detect the concentration of 48 cytokines in the supernatants of ARPE-19 cells incubated with each purified IgG. The culture supernatants of the ARPE-19 cells treated with PM IgG showed intense elevation of multiple cytokines, including pro-inflammatory cytokines (interleukin 9 [IL-9] and tumor necrosis factor beta [TNF-β]) and chemokines (macrophage inflammatory protein one beta [MIP-1b] and regulated upon activation normally T-cell expressed and secreted [RANTES]), compared with those treated with emmetropia IgG or simple HM IgG (ANOVA with LSD post hoc test, all *p* < 0.0001, Fig. 7*B*). These effects were all attenuated after removal of the anti-LIMS1 autoantibody (ANOVA with LSD post hoc test, all *p* < 0.0001, Fig. 7*B*).

## Discussion

High myopia, defined as a myopic refractive error with a spherical equivalent ≤ −6.00 D or AL ≥ 26.00 mm, is a major public health issue worldwide (1, 19). Thereinto, pathologic myopia, characterized by significant MMD, is the most detrimental subtype of high myopia. Previous studies have reported that it affects up to 3% of the world’s population (20). Pathologic myopia is distinctly different from high myopia, and has a significantly worse visual prognosis. The Beijing Eye Study showed that pathologic myopia was the second leading cause of low vision and blindness in Chinese adults (21), posing great social and economic burdens. Doctors previously thought that severe fundus lesions randomly appeared in some highly myopic patients. However, with the distinct severity of MMD, eye specialists are now more aware of the differences between pathologic myopia and simple high myopia. Indeed, they may be two entirely different subtypes, which should not be confused. Additionally, they may require different therapeutic strategies. However, the pathogenesis of pathologic myopia is complex and remains largely unknown.

MMD is characterized by the persistent and progressive degeneration of the neurosensory retina, RPE, and choroid throughout the lifetime. Previous studies have revealed a pro-inflammatory intraocular microenvironment in eyes with pathologic myopia, indicating the presence of chronic inflammation (6). These characteristics of pathologic myopia resemble the pathological changes in many autoimmune diseases. For example, rheumatoid arthritis, a common systemic autoimmune disease, is characterized by chronic synovial inflammation and irreversible joint damage, caused by anti-citrullinated protein antibodies (22, 23). Similarly, during the pathogenesis of autoimmune retinopathy, circulating anti-retinal autoantibodies persistently attack retinal cells inducing apoptosis, which is accompanied by cytokine release (8). These data suggest that persistent attack by autoantibodies is probably involved in the pathogenesis of pathologic myopia.

With proteomic tools, we have shown that the serum level of the anti-LIMS1 autoantibody is significantly increased in pathologic myopia and is a potential serum biomarker for this disease, supporting a previously unrecognized autoimmune aspect of the pathogenesis of pathologic myopia. It is noteworthy that even in patients at the earliest stage of pathologic myopia, that is MMD grade 2, the serum level of the anti-LIMS1 autoantibody is already clearly elevated, supporting its future clinical utility as an early biomarker of pathologic myopia. Different subtypes of autoantibody play distinct roles in the autoimmune process, and the predominant subclasses of the serum anti-LIMS1 autoantibody identified in pathologic myopia (IgG1/IgG2/IgG3) could provide further clues to the possible autoimmune responses involved in this disease. For example, IgG3 is a potent pro-inflammatory antibody, that activates the complement cascade and leads to overt inflammation (24). This may contribute to the pro-inflammatory intraocular microenvironment in pathologic myopia.

LIMS1, named senescent cell antigen-like domains 1, also known as PINCH1, is widely expressed in mammalian cells and plays an important role in cytoskeletal organization and cell-cell junctions (25, 26, 27). Previous studies have reported its involvement in kidney allograft rejection, an autoimmune disorder resulting from hypoxia-induced upregulation of the LIMS1 antigen in human kidney cells (28). However, no study has yet investigated its function in ocular diseases. In the present study, we found that the LIMS1 antigen is strongly expressed in RPE cells, suggesting that the RPE is the main pathogenic target of this autoantibody. RPE is known to act as an anatomical barrier, namely oBRB, between the neurosensory retina and the choroid (29). The treatment of RPE cells with IgG extracted from the sera of patients with pathologic myopia significantly impaired their barrier function, whereas the depletion of the anti-LIMS1 autoantibody attenuated this effect. Further investigation suggested that the anti-LIMS1 autoantibody disrupt the barrier function by disturbing the cytoskeletal organization and cellular tight junctions of RPE cells. Normally, the highly selective transcellular active-transport systems in the oBRB allow the close regulation of the specialized retinal microenvironment (30). Disruption of the oBRB is almost always the primary pathological change in retinal degenerative diseases, such as AMD (31, 32). This is followed by the death and dysfunction of photoreceptors and choriocapillaris (33), which result in irreversible macular degeneration and chorioretinal atrophy. Hudson *et al.* (34) showed that BRB leakage, caused by suppressed expression of tight junction proteins, can lead to macular degeneration in mice. Therefore, the disruption of the oBRB by the anti-LIMS1 autoantibody may cause the deterioration in the microenvironment of the photoreceptor layer, resulting in macular degeneration in eyes with pathologic myopia.

Further investigation suggested that the anti-LIMS1 autoantibody also induces a pro-inflammatory state in RPE cells and triggers the release of pro-inflammatory mediators, which is similar to the autoimmune pathogenesis of uveitis (35). In the present study, we have shown that the anti-LIMS1 autoantibody induces upregulation of ICAM-1, IL-9, TNF-β, MIP-1b, and RANTES in RPE cells. As previous studies have reported, the autoantibody promotes the upregulation of adhesion proteins by activating the nuclear factor kappa B pathway by binding to antigens on endothelial cells (36, 37). The expression of ICAM-1, an adhesion molecule, is probably upregulated when the anti-LIMS1 autoantibody binds to LIMS1 antigens, and initiates the recruitment of autoreactive lymphocytes, and helps them cross the RPE (35). Both IL-9 and TNF-β are known to be involved in the inflammatory response in many autoimmune diseases (38, 39, 40), and MIP-1b and RANTES could chemoattract monocytes/macrophages and T cells to the target site. The current evidence suggested that, the elevated expression of adhesion molecules, pro-inflammatory cytokines, and chemokines in RPE cells caused by anti-LIMS1 autoantibody attack, allows the recruitment of immune cells and the initiation of an inflammatory cascade. These, in turn, are responsible for subsequent inflammatory tissue damage during the pathogenesis of pathologic myopia.

Our study demonstrates that the serum anti-LIMS1 autoantibody is a potential biomarker for pathologic myopia, indicated by its close association with the grades of MMD. Its detrimental effects on RPE cells uncover a previously unrecognized autoimmune etiology for pathologic myopia, which may lead to a paradigm shift in the prediction, prevention, and treatment of pathologic myopia.

## Data Availability

All data needed to evaluate the conclusions in the paper are present in the paper and the supplementary materials, and microarray data will be publicly available online through the Gene Expression Omnibus database (GSE223434).

## Supplemental data

This article contains supplemental data.

## Conflict of interest

The authors declare that they have no known competing financial interests or personal relationships that could have appeared to influence the work reported in this paper.
