# Supplementary material for: Circulating Autoantibody Profiling Identifies LIMS1 as a Potential Target for Pathogenic Autoimmunity in pathologic Myopia
Source: Mol Cell Proteomics. 2024 May 9;23(6):100783. doi: 10.1016/j.mcpro.2024.100783 (PMC11215957; doi:10.1016/j.mcpro.2024.100783)
Supplement: Supplemental Data [file mmc1.docx]

**Fig. S1. Study design of identification of pathologic myopia-related autoantibody using a three-phase strategy.**


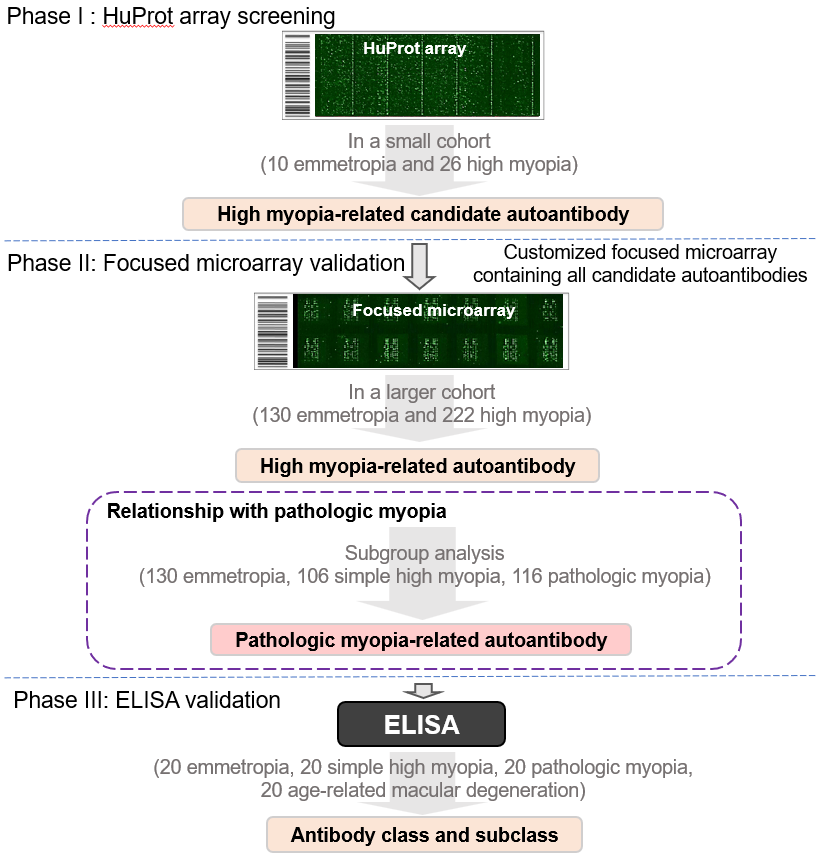


**Fig. S2. Expression of LIMS1 antigen on ARPE-19 cells after exposure to each purified IgG using immunofluorescence staining.**


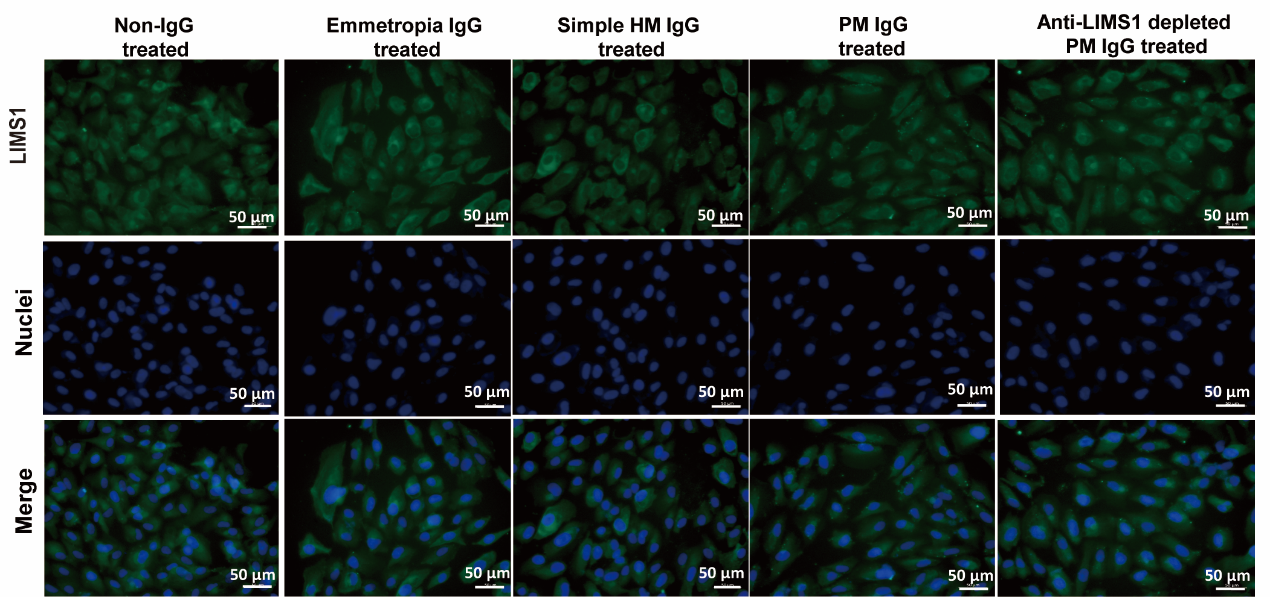


Immunofluorescent images of LIMS1 antigen staining in ARPE-19 cells after exposure to non-IgG, and purified IgG (500 μg/mL) extracted from the serum samples of subjects with emmetropia (emmetropia IgG), simple high myopia (simple HM IgG), pathologic myopia (PM IgG), or PM IgG depleted of anti-LIMS1 autoantibody (anti-LIMS1 depleted PM IgG).

**Fig. S3. Effect of each purified IgG on ARPE-19 cells using immunofluorescence staining.**


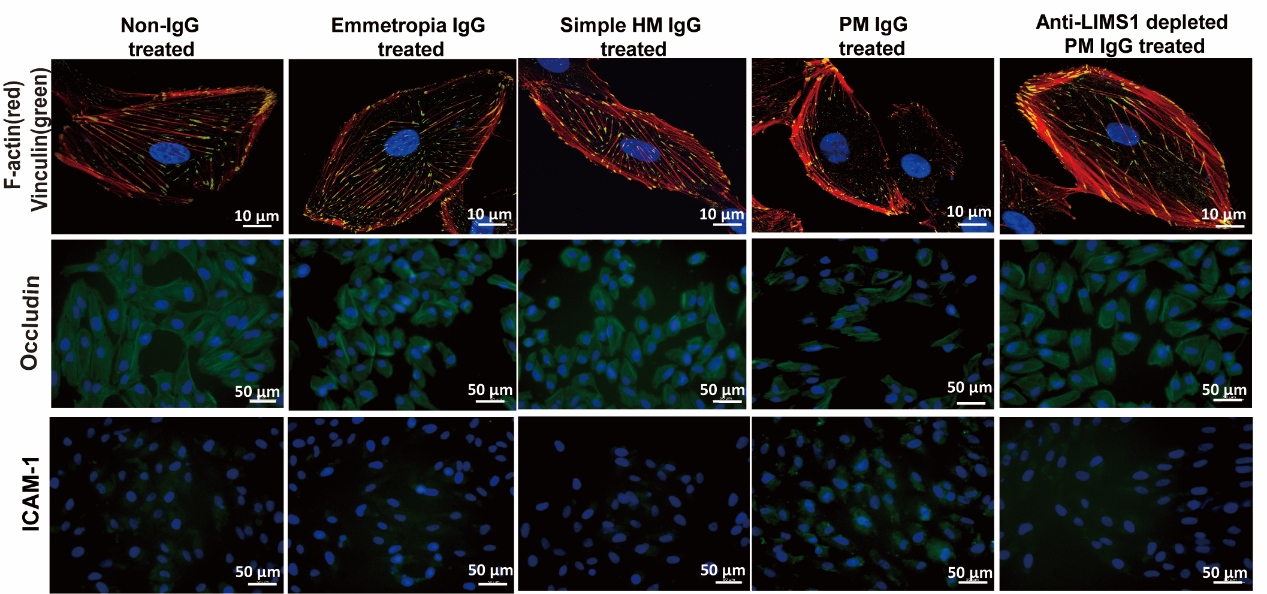


Immunofluorescent images of F-actin (red) and vinculin (green) (top), occludin (middle) and ICAM-1 (bottom) staining in ARPE-19 cells after exposure to non-IgG, and purified IgG (500 μg/mL) extracted from the serum samples of subjects with emmetropia (emmetropia IgG), simple high myopia (simple HM IgG), pathologic myopia (PM IgG), or PM IgG depleted of anti-LIMS1 autoantibody (anti-LIMS1 depleted PM IgG).**Table S1. Demographic characteristics of all participants in three steps.**

|  | Emmetropia | High myopia | | Age-related macular degeneration | P value |
| --- | --- | --- | --- | --- | --- |
|  |  | Simple high myopia | Pathologic myopia |  |  |
| **Phase I** |  |  |  |  |  |
| No. | 10 | 10 | 16 |  |  |
| Age, years | 63.7 ± 5.7 | 61.8 ± 6.5 | 60.8 ± 7.7 |  | 0.586 |
| Sex, male/female | 5/5 | 4/6 | 6/10 |  | 0.814 |
| Axial length, mm | 23.18 ± 0.74 | 28.76 ± 1.82 | 30.39 ± 2.20 |  | 0.000* |
| **Phase II** |  |  |  |  |  |
| No. | 130 | 106 | 116 |  |  |
| Age, years | 63.1 ± 7.8 | 60.8 ± 10.6 | 62.3 ± 10.6 |  | 0.179 |
| Sex, male/female | 56/74 | 58/48 | 50/66 |  | 0.134 |
| Axial length, mm | 23.19 ± 0.76 | 28.13 ± 1.66 | 30.06 ± 2.31 |  | 0.000* |
| **Phase III** |  |  |  |  |  |
| No. | 20 | 20 | 20 | 20 |  |
| Age, years | 62.9 ± 7.2 | 62.0 ± 8.8 | 63.8 ± 7.9 | 69.0 ± 8.6 | 0.039* |
| Sex, male/female | 12/8 | 9/11 | 10/10 | 8/12 | 0.626 |
| Axial length, mm | 22.97 ± 0.58 | 28.45 ± 1.89 | 29.83 ± 2.39 | 23.55 ± 0.68 | 0.000* |

Comparison among groups was analyzed using one-way analysis of variance (continuous data) or χ2 test (categorical data).

*Statistically significant (P < 0.05).

**Table S2. Positive rates of high myopia-related candidate autoantibodies detected by HuProt arrays during Phase I.**

| Autoantibody name  (IgG or IgM) | Positive rate in emmetropia  (n = 10) | Positive rate in high myopia  (n = 26) | P value |
| --- | --- | --- | --- |
|  |  |  |  |
| Anti-ZNF48 | 2/10 (20.0%) | 21/26 (80.8%) | 0.001 |
| Anti-HSPA1L | 0/10 (0.0%) | 12/26 (46.2%) | 0.015 |
| Anti-LIMS1 | 0/10 (0.0%) | 12/26 (46.2%) | 0.015 |
| Anti-ZNF154 | 0/10 (0.0%) | 12/26 (46.2%) | 0.015 |
| Anti-FAM168A | 0/10 (0.0%) | 12/26 (46.2%) | 0.015 |
| Anti-ZNF169 | 0/10 (0.0%) | 12/26 (46.2%) | 0.015 |
| Anti-SPA17 | 0/10 (0.0%) | 12/26 (46.2%) | 0.015 |
| Anti-ZIK1 | 1/10 (10.0%) | 15/26 (57.7%) | 0.022 |
| Anti-ZSCAN5A | 1/10 (10.0%) | 15/26 (57.7%) | 0.022 |
| Anti-NDEL1 | 2/10 (20.0%) | 17/26 (65.4%) | 0.024 |
| Anti-ATP8B5P | 2/10 (20.0%) | 17/26 (65.4%) | 0.024 |
| Anti-MRPL1 | 1/10 (10.0%) | 14/26 (53.8%) | 0.024 |
| Anti-Supt6h | 1/10 (10.0%) | 14/26 (53.8%) | 0.024 |
| Anti-PRMT7 | 0/10 (0.0%) | 9/26 (34.6%) | 0.039 |
| Anti-ENG | 0/10 (0.0%) | 9/26 (34.6%) | 0.039 |
| Anti-H2AFY2 | 0/10 (0.0%) | 9/26 (34.6%) | 0.039 |
| Anti-PDE4D | 0/10 (0.0%) | 9/26 (34.6%) | 0.039 |
| Anti-HLCS | 0/10 (0.0%) | 9/26 (34.6%) | 0.039 |

Comparison between groups was analyzed using Fisher’s exact test.

**Table S3. Signal intensity of high myopia-related candidate autoantibodies detected by customized focused microarrays during Phase II.**

| Autoantibody name | IgG | | |  | IgM | | |
| --- | --- | --- | --- | --- | --- | --- | --- |
|  | Fold change | P value | AUC |  | Fold change | P value | AUC |
| Anti-ZNF48 | 1.03 | 0.578 | 0.537 |  | 0.96 | 0.690 | 0.488 |
| Anti-HSPA1L | 1.05 | 0.603 | 0.520 |  | 0.86 | 0.143 | 0.450 |
| Anti-LIMS1† | 1.34 | 0.000* | 0.697 |  | 1.19 | 0.002 | 0.573 |
| Anti-ZNF154 | 1.01 | 0.497 | 0.607 |  | 1.02 | 0.032 | 0.590 |
| Anti-FAM168A | 1.09 | 0.115 | 0.542 |  | 1.01 | 0.919 | 0.524 |
| Anti-ZNF169 | 1.19 | 0.226 | 0.586 |  | 1.11 | 0.147 | 0.541 |
| Anti-SPA17 | 1.25 | 0.263 | 0.528 |  | 0.92 | 0.645 | 0.525 |
| Anti-ZIK1 | 1.04 | 0.348 | 0.590 |  | 1.05 | 0.324 | 0.518 |
| Anti-ZSCAN5A | 1.26 | 0.000* | 0.636 |  | 1.15 | 0.035 | 0.541 |
| Anti-NDEL1 | 0.93 | 0.499 | 0.512 |  | 1.11 | 0.215 | 0.545 |
| Anti-ATP8B5P | 1.24 | 0.051 | 0.537 |  | 1.06 | 0.045 | 0.543 |
| Anti-MRPL1 | 1.35 | 0.011 | 0.578 |  | 1.04 | 0.747 | 0.562 |
| Anti-Supt6h | 1.25 | 0.085 | 0.582 |  | 0.92 | 0.573 | 0.547 |
| Anti-PRMT7 | 0.79 | 0.220 | 0.492 |  | 1.09 | 0.408 | 0.541 |
| Anti-ENG | 1.09 | 0.110 | 0.551 |  | 1.00 | 0.950 | 0.526 |
| Anti-H2AFY2 | 1.14 | 0.148 | 0.561 |  | 1.05 | 0.583 | 0.570 |
| Anti-PDE4D | 1.02 | 0.080 | 0.549 |  | 1.03 | 0.246 | 0.511 |
| Anti-HLCS | 1.08 | 0.597 | 0.542 |  | 1.23 | 0.211 | 0.534 |

Comparison between groups was analyzed using Students’s t-test. Receiver operating characteristic (ROC) analysis was performed to identify the area under the ROC curve (AUC).

*P < 0.00139 (0.05/36) after Bonferroni correction was considered statistically significant.

†The autoantibody with a fold change of signal intensity > 1.3, P value < 0.00139, and AUC > 0.6 between emmetropia and high myopia groups was identified as high myopia-related autoantibody.
